# East Texas Medico-Chirurgical Society, Second Semi-Annual Session, Palestine, Texas, November 26th to 30th, 1900

**Published:** 1900-11

**Authors:** 


### East Texas Medico-Chirurgical Society, Second Semi-Annual Session, Palestine, Texas, November 26th to 30th, 1900.

PROGRAM.

*First Day, Thursday, November 29th, 1900, at 2 p. m.*

1. Call to Order by Chairman Committee on Arrangements.
2. Invocation....................Rev. I. M. Merlinjones, D. D.
3. Address of Welcome.................Hon. T. M. Campbell.
4. Response by President...........Sam R. Burroughs, M. D.
5. Roll Call.
6. Reading of Minutes.
7. Applications for Membership.
8. Reports of Committees.
9. Reading of Papers—Section on Pediatrics:
   The Child's Second Summer..Dr. S. R. Burroughs, Buffalo.
10. Adjourn until 8:00 o'clock p. m.

*First Day, Evening Session, 8 p. m.*

1. Reading Papers—Section on General Medicine:
   Typhoid Fever.................Dr. M. E. McClure, Alto.
   Malaria......................Dr. E. W. Link, Palestine.
   Pneumonia..................Dr. C. S. Lane, Jacksonville.
   Catarrhal Fever..............Dr. J. M. Colley, Palestine.
2. Banquet.
3. Adjourn until 9:00 o'clock a. m.

*Second Day, Friday, November 30th, 1900, 9 a. m.*

1. Executive Business.
2. Reading Papers—Section on Surgery:
   Surgical Emergencies...........Dr. J. S. Wiggins, Rusk.
   Abdominal Surgery........Dr. A. L. Hathcock, Palestine.
   Head Injuries.................Dr. Wm. Honey, Buffalo.
   Gonorrhea...................Dr. J. H. Paxton, Elkhart.
   Section on Obstetrics and Gynecology:
   Pelvic Abscess...............Dr. R. M. Dunn, Palestine.
   Placenta Previa..............Dr. E. B. Stokes, Crockett.
   Injuries to Pelvic Floor and Their Treatment........
   ......................Dr. J. S. Wooters, Crockett.
   Puerperal Eclampsia..Dr. M. D. McCarty, Porter Springs.
3. Unfinished and Miscellaneous Business.
4. Adjournment.

COMMITTEES.

*Arrangement.*—Dr. A. L. Hathcock, Chairman; Dr E. W. Link, Dr. J. H. Evans.

*Program.*—Dr. E. B. Parsons, Chairman; Dr. H. R. Link, Dr. R. L. McMeans.

Physicians are invited to be present.

